# A case of limb shaking transient ischaemic attack due to internal carotid artery dissection: an unusual presentation of fibromuscular dysplasia

**DOI:** 10.1186/s12883-023-03130-9

**Published:** 2023-03-01

**Authors:** Lei Si, Jing Tu, Hui Lei, Liya Ji, Zhiyong Zhang, Zhiqin Liu

**Affiliations:** 1 Department of Neurology, Xi’an Central Hospital, Xi’an, Jiaotong University School of Medicine, Xi’an 710003, Shaanxi, China; 2 Xi’an Medical University, Xi’an 710021, Shaanxi, China; 3 Department of Neurology, Beijing Geriatric Hospital, Beijing, 100095 China

**Keywords:** Limb shaking TIA, Stroke, Fibromuscular dysplasia, Dissection, Carotid artery

## Abstract

**Background:**

Fibromuscular dysplasia (FMD) has a high prevalence of associated nontraumatic carotid artery dissection, which could further result in transient ischaemic attack (TIA) or stroke. Limb shaking TIA is an unusual form of TIA that is commonly discribed in elderly patients with atherosclerotic backgrounds, while there are limited data about it in patients with FMD. Furthermore, discussions of limb shaking TIA in nonelderly patients are scarce.

**Case presentation:**

An Asian 47-year-old female presented with intermittent involuntary movement of the left upper limb accompanied by neck torsion. The episode stopped soon after changing to the supine position. On native source images of time-of-flight magnetic resonance angiography (TOF-MRA), the right internal carotid artery showed a "dual lumen sign" with an intimal flap. On contrast-enhanced magnetic resonance angiography and sagittal black-blood T1WI, an intravascular haematoma with irregular lumen stenosis was observed, which overall indicated right internal carotid artery dissection. Digital subtraction angiography showed the characteristic “string-of-beads” appearance in the left internal carotid artery, and the presence of this sign pointed to the diagnosis of FMD. The patient was finally diagnosed with limb shaking TIA due to internal carotid dissection with fibromuscular dysplasia. The patient was prescribed dual anti-platelet therapy. The limb shaking vanished soon after admission with no reoccurrence in the three-month follow-up.

**Conclusions:**

This case demonstrates that limb shaking TIA can present in patients with FMD. Limb shaking TIA in nonelderly patients can be caused by multiple diseases, and more detailed patient guidance is required in clinical practice.

**Supplementary Information:**

The online version contains supplementary material available at 10.1186/s12883-023-03130-9.

## Background

Fibromuscular dysplasia (FMD) is a nonatherosclerotic, noninflammatory vascular disease of small- and medium-sized arteries. When FMD involves cervical arteries (carotid and vertebral arteries), it presents as stenosis, occlusion, aneurysm, or dissection that could further lead to acute cerebrovascular events, which are even seen as the first remarkable clue for the clinical diagnosis of FMD [1]. Thus, timely identification of the manifestation of acute cerebrovascular events in FMD is urgently needed. Limb shaking is an unusual presentation in transient ischaemic attack (TIA) and is characterized by brief, recurrent involuntary movements involving the extremities and possibly accompanied by other neurological deficits. While usually occurring in elderly patients with severe atherosclerotic steno-occlusive diseases, limb shaking TIA was also reported in younger patients with multiple diseases, such as Moyamoya disease, spontaneous internal carotid (ICA) dissection and middle cerebral artery (MCA) dissection [2–4]. To our knowledge, limb shaking TIA caused by FMD has rarely been reported.

Here, we reported a middle-aged Asian female diagnosed with limb shaking TIA secondary to FMD-related ICA dissection, and this case provided a novel link between this unusual presentation and FMD. Furthermore, we discussed the diseases that could cause limb shaking TIA in nonelderly patients (under 60 years old). This study followed the CARE guidelines (Suppl.)

## Case presentation

**Fig. 1:**
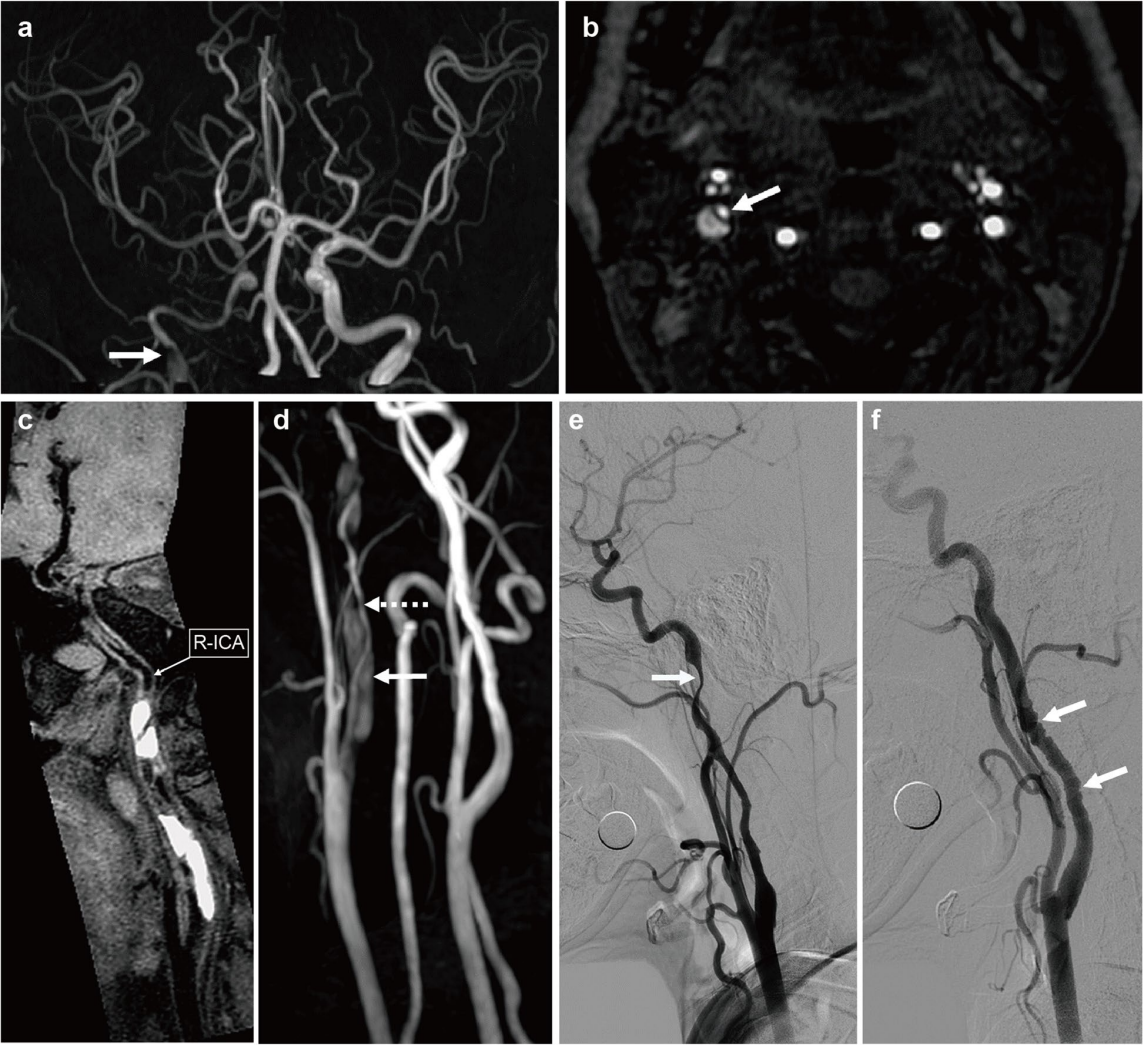
Imaging at the first visit. **a** Brain time-of-flight imaging magnetic resonance angiography (TOF-MRA) showed stenosis of the right internal carotid artery (ICA) in the C1 segments with a focus filling defect (white arrow); the lumen of the right ICA in the C2 and C3 segments had become narrow; **b** On TOF-MRA, the native source image showed that the right ICA was divided into two lumens by an intimal flap; **c** Sagittal imaging (black-blood T1WI) showed intimal flaps within the lumen and a hyperintense intravascular haematoma in the right ICA; **d** Cervical TOF-MRA showed a narrowing lumen of the C1 segment of the right ICA (white dotted line arrow) and an intravascular haematoma (white arrow); **e** Digital subtraction angiography (DSA) showed a “line-like” sign at the distal portion of the C1 segment of the right ICA (white arrow). **f** DSA showed the “string-of beads” appearance in the distal part of the C1 segment of the left ICA (white arrow).

**Fig. 2:**
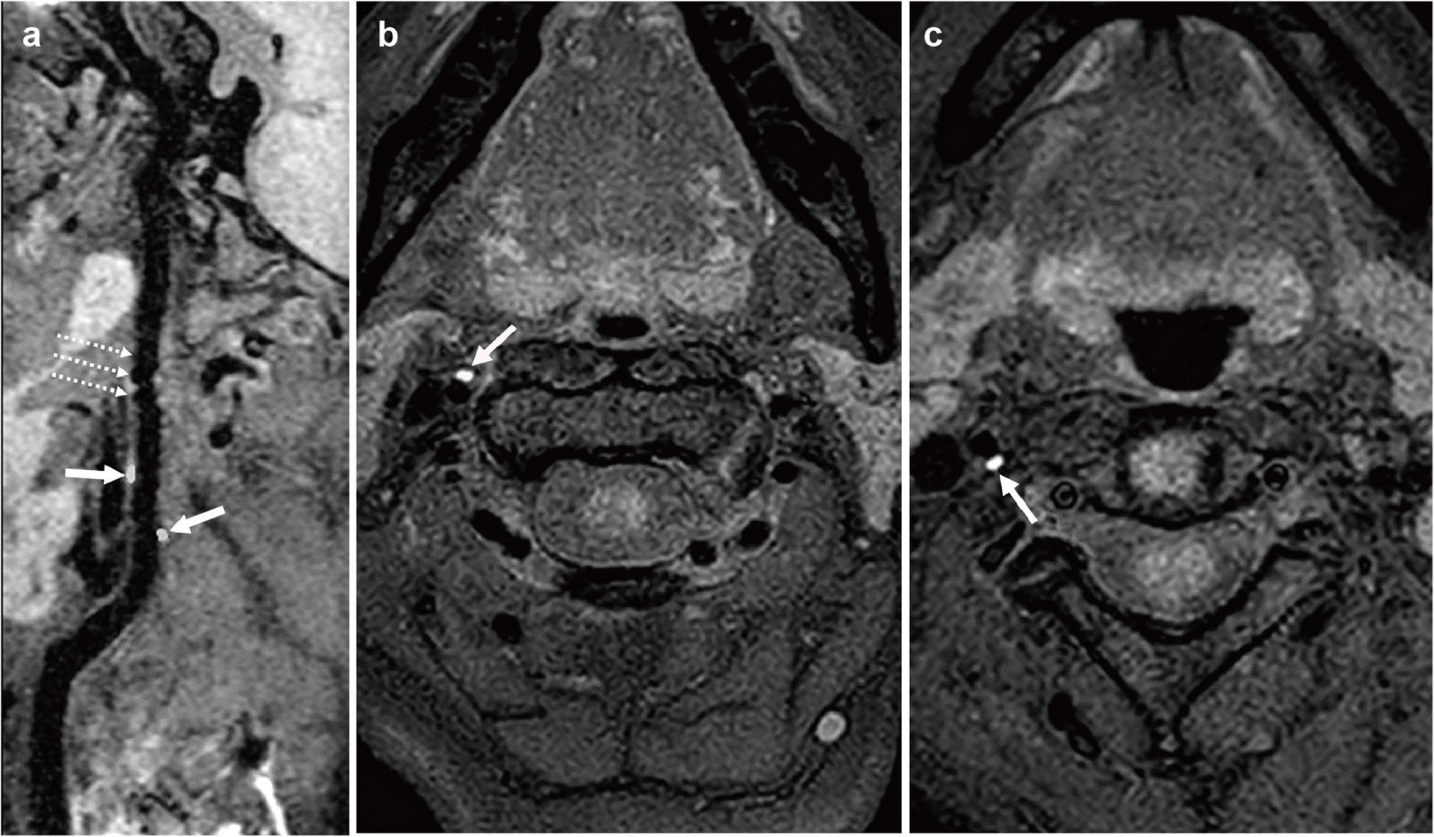
3-month follow-up imaging. **a** High resolution magnetic resonance imaging of the vascular wall (black-blood T1WI) showed the "string-of-beads” appearance (white dotted line arrow) and two hyperintense residual intravascular haematomas (white arrow) in the vascular wall of the right ICA; **b** and **c**. Axial imaging showed a hyperintense residual intravascular haematoma of the right ICA.

A 47-year-old female Asian with intermittent involuntary movements of the left upper limb was admitted to our facility. The limb shaking was coarse, rhythmic and flapping wing-like, accompanied by neck torsion while sparing the face and trunk (Supp2.Video.1). The first episode occurred 14 h before admission when the patient had arisen from supine position, and another episode also occurred when a similar postural change occurred soon after admission. Both episodes lasted 2–3 min and stopped 20 s after the patient changed back to the supine position. The patient had no consciousness disturbances, urinary incontinence, dystonia posture or tongue biting during the attacks. The patient reported a nonremarkable personal history and had no history of epilepsy, stroke, hypertension and other cerebrovascular disease risk factors, together with no report of any major trauma, surgery or any other events in the previous 6 months. Physical examination showed no neurological deficits except mild weakness in her left limb (Medical Research Council scale: 4). The neuropsychological evaluation was normal. Cardiological evaluations, including echocardiography, electrocardiography and cardiac biochemical markers (pro-brain natriuretic peptide, creatine kinase MB isoenzyme, troponin T), were without apparent abnormalities. Video-electroencephalogram and diffusion-weighted imaging were performed immediately after admission with no remarkable findings, whereas time-of-flight imaging magnetic resonance angiography (TOF-MRA) revealed multiple irregular stenoses of the right ICA accompanied by multiple filling defects (Fig. 1a). On native source TOF-MRA images, a "dual lumen sign" with an intimal flap was detected in the right ICA (Fig. 1b). Spontaneous right ICA dissection was suspected. Before further investigation, statin therapy with dual antiplatelet therapy (hydroclopidogrel, 75 mg per day, combined with aspirin, 100 mg per day) was administered for three days with no recurrence of limb shaking. More examinations were performed afterwards. On contrast-enhanced magnetic resonance angiography (CE-MRA) and sagittal black-blood T1WI, an intravascular haematoma (Fig. 1c) with irregular lumen stenosis was observed, which overall indicated right ICA dissection (Fig. 1d). The fact that TIA occurred in a relatively young female with no cerebrovascular risk factors led us to evaluate the cervical and cerebral vasculature more thoroughly and precisely. Thus, digital subtraction angiography (DSA) was performed. The DSA further confirmed the dissection because a "line-like" change was observed (Fig. 1e), while the renal artery was normal. Meanwhile, in the left ICA, we detected a “string-of-beads” appearance (Fig. 1f), which led to a suspicion of FMD. Although the ICA dissection related to the limb shaking TIA was not at the same site as the “string-of-beads” appearance, we still highly suspected that the ICA dissection was probably secondary to FMD. Sustained dual anti-platelet therapy was prescribed after discharge, and we suggested that she measure her blood pressure daily. Three months later, the patient returned to our department with no recurrence of limb shaking or neurological deficits. High-resolution magnetic resonance vessel wall imaging (HRMR-VWI) was performed to detect pathological changes in the vessel wall. The previous dissection in the right ICA had been mostly resolved, and only a few spotted intramural haematomas were left (Fig. 2). Notably, a characteristic "string-of-beads” appearance was observed on the right ICA at this time (Fig. 2a). Considering the overall clinical data, the patient was diagnosed with limb shaking TIA due to ICA dissection with FMD.

## Discussion and conclusions

Here, we present a case of limb shaking TIA with FMD, which has not been reported before. FMD is an arterial dysplasia disease that has a propensity to occur in many vascular beds. The most commonly involved arteries were the renal and internal carotid arteries with a typical “string-of-beads” appearance on vessel imaging. Apart from nonspecific symptoms such headache, pulsatile tinnitus and dizziness, TIA or stroke may be the first reason for FMD patients to visit the hospital [5]. Limb shaking is an underrecognized symptom of TIA, and there is limited understanding of its anatomical and pathophysiologic mechanisms. In this case, the limb shaking symptom was hyperkinetic and usually lasted less than 5 min. Patients with limb shaking TIA can be misdiagnosed with seizures because of the similar brief, stereotypical episode, especially those patients who are younger. Current studies mostly attribute limb shaking TIA to cerebral hypoperfusion secondary to a postural change, exercising, or sudden neck extension, which commonly occurs in elderly patients with atherosclerotic stenosis or occlusion of the ICAs or middle cerebral arteries. However, in patients under the age of 60 years, many other diseases can cause limb shaking TIA without an atherosclerotic background.

Moyamoya disease was the most widely investigated vasculopathy in nonelderly patients with limb shaking TIA [3, 6], which was highly in accordance with the age distribution of Moyamoya disease [3]. The published triggers were mostly in coincidence with those in elderly patients, such as exercise and hyperventilation, while some unique triggers were reported, such as eating hot spicy food, singing, sneezing, prolonged crying, emotional stress, and smoking [3]. Compared to limb shaking TIA patients with atherosclerotic backgrounds, whose limb shaking usually vanishes after treatment [7], patients with Moyamoya disease experience more controversial outcomes after treatment. In 2021, a study about limb shaking TIA with Moyamoya disease showed that all the patients in their series still had limb shaking attacks, even after 7–36 months of follow-up [3]. Notably, other published case reports and series suggested that patients who underwent revascularization surgery had full resolution of limb shaking during 3–12 months of follow-up [3]. These controversial results might be due to different therapy choices. Although stenosis in the ICA or intracranial arteries was detected in all patients in the 2021 series, none of them received revascularization surgery because of failure to access consent. Conservative therapy with oral anti-platelets was prescribed in all patients with no further vessel imaging tests or perfusion studies to evaluate the effectiveness. However, surgical revascularization is recommended for patients with symptomatic Moyamoya disease [8, 9], and revascularization may result in better outcomes than conservative therapy in limb shaking TIA patients with Moyamoya disease. Moyamoya disease has a progressive vascular pathology [10, 11]. The intracranial artery involvement worsens with disease progression and can lead to repeated ischaemic strokes or haemorrhage, even after surgical treatment [12]. Other than the different therapies the patients had, controversial results can also occur due to another reason. Some patients in the 2021 series were children who might have difficulty maintaining therapeutic levels, which compromises the therapeutic effectiveness.

Spontaneous artery dissection was another reason for limb shaking TIA in nonelderly patients. Liu reported a 26-year-old male with limb shaking TIA caused by a spontaneous right MCA dissection [2]. The identified vascular risk factor was smoking history. The attacks occurred during postural changes and would last for a few seconds. A Turkish study reported a 47-year-old male patient with hyperhomocysteinemia and hyperlipidaemia who was diagnosed with limb shaking TIA due to a spontaneous ICA dissection [4]. A similar case was documented in a 41-year-old male [13]. Rare aetiologies were also reported. A novel case of limb shaking TIA associated with PHACE, an abbreviation for malformations of the posterior fossa, facial haemangiomas, arterial anomalies, cardiac anomalies, and abnormalities of the eye, was reported [14]. The 23-year-old female patient visited the hospital because of suffering from an insidious onset of hemiparesis since infancy. Her limb shaking was thought to be induced by the subclavian steal phenomenon. Brain MRI revealed multiple hypoplastic arteries, including the left ICA, MCA, and vertebral artery. Extracranial Doppler ultrasonography revealed stenosis of the left subclavian artery, which together with hypoplasia of the ICA led to an attack of limb shaking TIA in this patient. Apart from the congenital vasculopathy, an ictal procedure-induced limb shaking TIA case was mentioned in 2015. A 54-year-old male with a non-remarkable medical history underwent elective balloon test occlusion because of an aneurysm in the distal C1-portion of the right ICA [15]. However, 9 min after insufflation, the patient developed limb shaking of the left arm and leg with preserved strength, together with left-side body paraesthesia. However, there was still a nonnegligible quantity of nonelderly patients with limb shaking TIAs who had ICA or MCA stenosis and occlusion with atherosclerotic backgrounds. However, these patients were usually enrolled in large cohort studies without detailed clinical data, which makes it impossible to further discuss here. Largely, the therapy choices made for these patients were no different from those in elderly patients.

The identification of limb shaking as a symptom of TIA presents a great challenge, especially in younger patients, because hyperkinetic movement could be easily misinterpreted as a seizure or a metabiotic disturbance at first, which are two differential diagnoses that are much more commonly considered than TIA. Our case and previous studies indicated that limb shaking has been a neglected symptom in nonelderly TIA patients. There were some clinical clues to help make the differential diagnosis. First, collecting detailed medical and related personal histories with eyewitness descriptions could provide usable hints. Limb shaking TIA usually occurs with a participating factor, such as hyperventilation, postural change and exercise, which may compromise cerebral perfusion. In our case, the episodes were caused by a particular postural change stereotypically, which indicated a possible posture-related perfusion change. Second, the importance of angiography should be stressed in nonelderly patients with limb shaking TIA for evaluating for any potential pathologic conditions, such as arterial dysplasia. The use of this technique not only helps to manage ischaemic events but also provides a great chance for aetiological diagnosis. Compared with conventional angiography, such as DSA, TOF-MRA, and computed tomography angiography, which focus on the vessel lumen, HRMR-VWI can also detect the vessel wall configuration and inflammatory changes [16]. It has been applied in many vascular diseases, such as vasculitis, intracranial vasculopathies, Moyamoya disease and arterial dissection [16–19]. In nonelderly patients with cerebrovascular events, HRMR is beneficial for determining the aetiology diagnosis, especially for possible vasculopathy, which may be without vessel lumen narrowing. Our case suggests that limb shaking TIA in nonelderly patients may be the first presentation of a systematic disease. However, the final diagnosis may demand time and patience. Here, the diagnosis of FMD was finally achieved at the second visit. Rigorous diagnostic workup was needed to determine the potential disease. More disease-specific guidance is necessary to prevent severe complications afterwards. We suggested that our patient undergo daily blood pressure measurement and regular renal artery Doppler ultrasound to evaluate possible renal artery involvement in the future. Furthermore, long-term follow-up will also be suggested to patients to help manage the disease. In this case, we failed to perform a longer follow-up beyond the patient’s last visit.

Our study demonstrated the heterogeneity of the disease associated with limb shaking TIA. When diagnosing intermittent limb movement in nonelderly patients, limb shaking TIA should be taken into consideration, and detailed patient guidance is necessary.

## Supplementary Information

**Additional file 1. Supp2. Video 1.** Video of our patient’s attack after admission. The patient presented with coarse, rhythmic and flapping wing-like left arm shaking, accompanied by neck torsion while sparing the face and trunk. The attack occurred when the patient arose from the supine position and was sustained approximately 20 seconds after changing into the supine position (the recording started after the patient changed into the supineposition, but the remission of the attack unfortunately failed to be recorded). The file format is mp4.

## Data Availability

Not applicable.
